# Deep learning and conventional hip MRI for the detection of labral and cartilage abnormalities using arthroscopy as standard of reference

**DOI:** 10.1007/s00330-025-11546-9

**Published:** 2025-04-16

**Authors:** Alexander W. Marka, Felix Meurer, Vanessa Twardy, Markus Graf, Kilian Weiss, Marcus R. Makowski, Dimitrios C. Karampinos, Jan Neumann, Klaus Woertler, Ingo J. Banke, Sarah C. Foreman

**Affiliations:** 1 Department of Diagnostic and Interventional Radiology, School of Medicine and Health & Klinikum rechts der Isar, Technical University of Munich, Munich, Germany; 2 Musculoskeletal Radiology Section, School of Medicine and Health & Klinikum rechts der Isar, Technical University of Munich, Munich, Germany; 3 Clinic of Orthopedics and Sports Orthopedics, Klinikum rechts der Isar, Technical University of Munich, Munich, Germany; 4 Philips GmbH, Hamburg, Germany; 5 Department of Diagnostic and Interventional Neuroradiology, School of Medicine and Health & Klinikum rechts der Isar, Technical University Munich, Munich, Germany

**Keywords:** Arthroscopy, Hip joint, Femoracetabular impingement, Magnetic resonance imaging, Deep learning

## Abstract

**Objectives:**

To evaluate the performance of high-resolution deep learning-based hip MR imaging (CSAI) compared to standard-resolution compressed sense (CS) sequences using hip arthroscopy as standard of reference.

**Methods:**

Thirty-two patients (mean age, 37.5 years (± 11.7), 24 men) with femoroacetabular impingement syndrome underwent 3-T MR imaging prior to hip arthroscopy. Coronal and sagittal intermediate-weighted TSE sequences with fat saturation were obtained using CS (0.6 × 0.8 mm) and high-resolution CSAI (0.3 × 0.4 mm), with 3 mm slice thickness and similar acquisition times (3:55–4:12 min). MR scans were independently assessed by three radiologists and a hip arthroscopy specialist for labral and cartilage abnormalities. Sensitivity, specificity, and accuracy were calculated using arthroscopy as reference standard. Statistical comparisons between CS and CSAI were performed using McNemar’s test.

**Results:**

Labral abnormality detection showed excellent sensitivity for radiologists (CS and CSAI: 97–100%) and the surgeon (CS: 81%, CSAI: 90%, *p* = 0.08), with 100% specificity. Overall cartilage lesion sensitivity was significantly higher with CSAI versus CS (42% vs. 37%, *p* < 0.001). Highest sensitivity was observed in superolateral acetabular cartilage (CS: 81%, CSAI: 88%, *p* < 0.001), while highest specificity was found for the anteroinferior acetabular cartilage (CS and CSAI: 99%). Sensitivity was lowest for the assessment of the anteroinferior and posterior acetabular zones, and inferior and posterior femoral zones (CS and CSAI < 6%).

**Conclusion:**

CS and CSAI MR imaging showed excellent diagnostic performance for labral abnormalities. Despite CSAI’s improved cartilage lesion detection, overall diagnostic performance for cartilage assessment remained suboptimal.

**Key Points:**

***Question*** *Accurate preoperative detection of labral and cartilage lesions in femoroacetabular impingement remains challenging, with current MRI protocols showing variable diagnostic performance.*

***Findings*** *High-resolution deep learning-based and standard-resolution compressed sense MRI demonstrate comparable diagnostic performance, with high accuracy for labral defects but limited sensitivity for cartilage lesions.*

***Clinical relevance*** *Current MRI protocols, regardless of resolution optimization, show persistent limitations in cartilage evaluation, indicating the need for further technical advancement to improve diagnostic confidence in presurgical planning.*

**Graphical Abstract:**

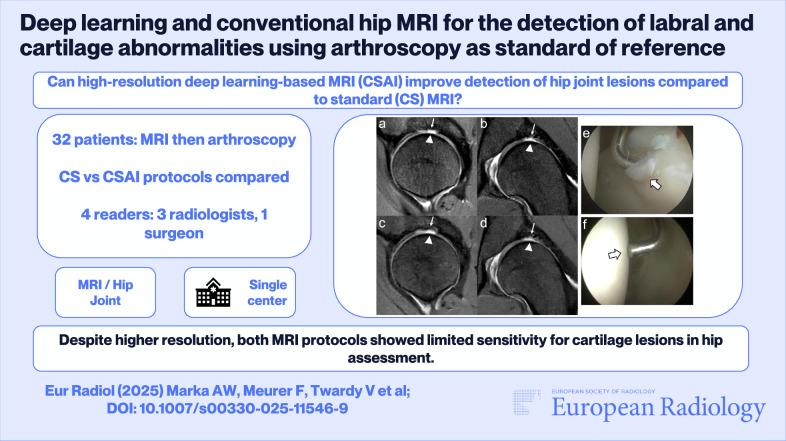

## Introduction

The accurate assessment of hip joint pathologies remains a significant challenge due to the curved anatomy and tight proximity of opposing articular surfaces [1–5]. The thin hyaline cartilage layers of the acetabulum and femoral head, measuring between 0.8 mm and 3.0 mm in healthy individuals, further complicate this evaluation, making it particularly difficult to detect small-scale pathologies [4, 6, 7]. Femoroacetabular impingement syndrome (FAIS) is a motion-related disorder characterized by abnormal contact between the femoral head-neck junction and the acetabular rim, leading to various degrees of chondrolabral damage and progressive hip pain [8]. Early detection of cartilage and labral abnormalities is crucial, as timely intervention with hip arthroscopy can significantly reduce the risk of onset and progression of hip osteoarthritis [9].

Magnetic resonance (MR) imaging is considered the gold standard for evaluating hip joint pathologies due to its superior soft-tissue contrast. Standard protocols typically include T1-weighted, T2-weighted, and fat-suppressed sequences. However, these conventional techniques often lack the spatial resolution required to effectively detect subtle labral and cartilage abnormalities. Recent advancements in MR imaging have focused on optimizing spatial resolution and tissue contrast while mitigating the prolonged acquisition times typically associated with high-resolution imaging. Compressed Sense (CS) accelerates image acquisition by undersampling k-space data and employing iterative image reconstruction algorithms to maintain diagnostic quality [10–12]. Building upon this, Compressed Sense Artificial Intelligence (CSAI) frameworks integrate parallel imaging, CS, and deep learning techniques to further enhance image quality and significantly reduce acquisition times without compromising diagnostic performance [13–16]. These developments are promising for improving the visualization of small anatomical structures and the detection of subtle pathologies in the hip joint [12, 14, 16, 17].

Therefore, the aim of this study was to evaluate the performance of high-resolution deep learning-based CSAI MR imaging protocols compared to standard-resolution CS protocols for the detection of labral and cartilage abnormalities in patients with FAIS using hip arthroscopy as standard of reference.

## Materials and methods

### Subject selection

Patients were prospectively enrolled between January and May 2022. Prior to participation, all individuals gave informed consent, and the study received approval from the local institutional review board (Protocol Number: 3/22 S). All patients were referred by our Clinic of Orthopedics and Sports Orthopedics with symptomatic FAIS [18]. Inclusion criteria included (1) age 18–65 years, (2) clinical diagnosis of FAIS based on positive impingement testing (FADIR test), and (3) hip pain for > 3 months. Exclusion criteria were: (1) prior hip surgery, (2) advanced osteoarthritis (Tönnis grade > 1), (3) inflammatory arthropathy, (4) hip dysplasia, and (5) MRI contraindications including pacemakers, implanted electronic devices, or pregnancy. Patients meeting study inclusion criteria underwent hip arthroscopy within 3 months of MR examination. Surgical intervention was indicated based on a combination of clinical symptoms, physical examination findings, and imaging results, following standard clinical practice guidelines for FAIS management [18].

### Image acquisition

All examinations were performed on a 3-T MR scanner (Ingenia Elition; Philips Healthcare) using a combination of a 12-channel posterior and a 16-channel anterior coil. The examination protocol included the following 2D sequences: sagittal and coronal intermediate-weighted (IM) turbo spin echo (TSE) sequences with spectral presaturation with inversion recovery (SPIR) for fat saturation. All sequences were obtained two times: (1) with an acceleration factor of 1–1.2 reconstructed using CS with a standard resolution of 0.6 × 0.8 mm and (2) with an acceleration factor of 2.3–3.3 reconstructed using CSAI with a high resolution of 0.3 × 0.4 mm. Both had a slice thickness of 3 mm and were acquired in the same timeframe of 3 min and 55 s to 4 min and 12 s. Further details of the imaging protocols are shown in Table 1.

**Table 1:** Sequence parameters of the sequences acquired using CS and CSAI

Pulse sequence	Sag IM FS TSE		Cor IM FS TSE	
	CS	CSAI	CS	CSAI
TR (ms)	2500	2500	1800	1800
TE (ms)	30	30	30	30
Echo train length (ETL)	11	10	10	10
Acq. resolution (mm)	0.6 × 0.8	0.3 × 0.4	0.6 × 0.8	0.3 × 0.4
Slice thickness (mm)	3	3	3	3
Field of view (mm)	160 × 160	160 × 160	190 × 170	190 × 170
Number of slices	22	22	22	22
Signal averages	1.5	2	1.5	2
Acceleration factor	1.2	3.3	1	2.3
Scan time (min)	3:55	3:55	3:54	4:12

Image reconstruction was based on a convolutional neural network (CNN), termed Adaptive-CS-Network, which integrates and improves the conventional CS algorithm as presented by Pezzotti et al [19]. Inspired by the iterative shrinkage-threshold algorithm (ISTA) presented by Zhang et al [20], the Adaptive-CS-Network incorporates multiscale sparsification in a problem-specific learnable manner. It combines a CNN-based sparsifying approach with compressed sense for image reconstruction, ensuring data consistency and integrating domain-specific knowledge. Unlike Pezzotti’s model, the Adaptive-CS-Network in this study replaces the wavelet transform with a CNN as a sparsifying transform in the compressed sense algorithm while preserving domain-specific knowledge and maintaining data consistency. Additionally, our network was pre-trained on around 740,000 sparsifying MR images, covering both 1.5-T and 3-T images across various anatomies and contrasts. The algorithm has been optimized for execution on standard reconstruction hardware and is commercially available (SmartSpeed, Philips Healthcare). In this manuscript, sequences reconstructed using the Adaptive-CS-Network are referred to as CSAI.

### Image analysis

**Fig. 1:**
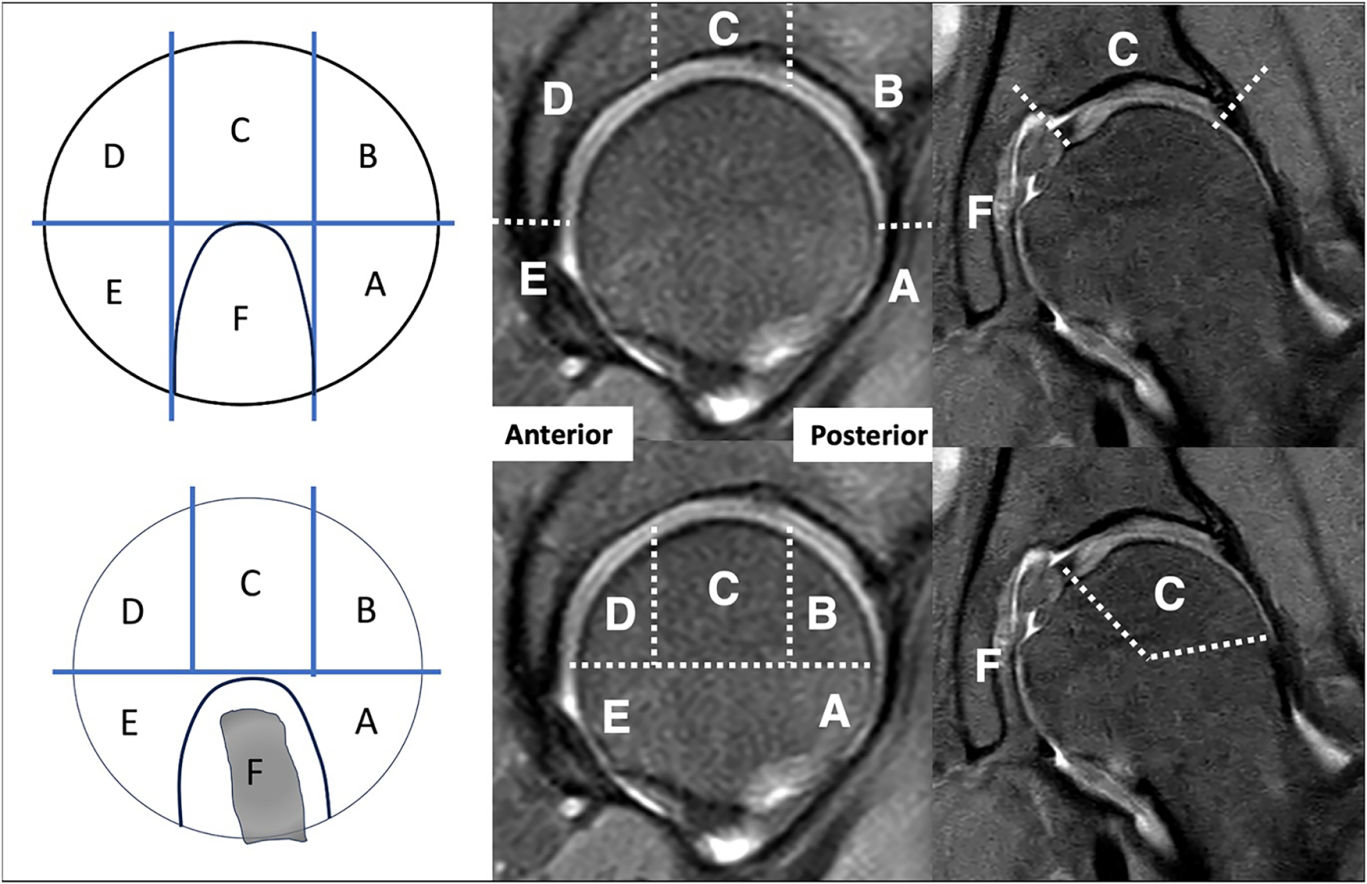
Sagittal diagram (left) and high-resolution sagittal (middle) and coronal (right) IM-weighted TSE sequences with SPIR of acetabular (top row) and femoral (bottom row) cartilage zones adapted from the Geographic Zone Method by Ilizaliturri et al [21], modified by Griffin et al [22, 23]. For the acetabulum, anterior-inferior is zone E, anterior-superior is zone D, mid-superior is zone C, posterior-superior is zone B, and posterior-inferior is zone A. For the Femur, central is zone A, posterior is zone B, superolateral is zone C, anterior is zone D, and inferior is zone E. Zone F is the acetabular notch as well as the fovea capitis femoris that contains the ligamentum capitis femoris. IM, intermediate-weighted; TSE, turbo spin echo; SPIR, spectral presaturation with inversion recovery

Four readers independently assessed all MR images, maintaining a four-week interval between readings of standard-resolution CS and high-resolution CSAI datasets to mitigate recall bias. Reading was performed by a general radiology resident (S.C.F., with 4 years of experience), a musculoskeletal (MSK) radiology fellow (F.M., with 8 years of experience), a fellowship-trained MSK radiologist (J.N., with 12 years of experience), and an orthopedic surgeon specialized in hip arthroscopy (I.J.B., with 20 years of experience). Readers were blinded to all clinical information and intraoperative findings to prevent bias. The images were assessed for the presence (1) or absence (0) of abnormalities of the acetabular labrum as well as presence or absence of acetabular and femoral cartilage lesions in six distinct zones (A–F) using the Geographic Zone Method by Ilizaliturri et al [21], modified by Griffin et al [22, 23], as depicted in Fig. 1. Labral abnormalities were defined as present if one or more of the following MRI findings were identified by raters: (1) irregular labral morphology with intrasubstance signal heterogeneity, (2) partial or complete detachment from the acetabular rim, (3) labral hypertrophy with internal signal alteration, or (4) complex tear patterns with labral fragmentation [24]. Cartilage damage was rated as present if any of the following MRI findings were detected: (1) focal or diffuse signal intensity alteration, (2) reduced cartilage thickness compared to adjacent normal cartilage, (3) surface irregularity or delamination, or (4) full-thickness cartilage loss with or without associated subchondral bone changes [25]. Acetabular and femoral Zone F were excluded from cartilage assessment. Acetabular Zone F comprises the acetabular fossa, which predominantly contains fibrofatty tissue and lacks hyaline cartilage [26]. Femoral Zone F corresponds to the attachment site of the ligamentum teres [26].

### Hip arthroscopy

Following MR imaging, surgical intervention for FAIS was performed by a single orthopedic surgeon specializing in hip arthroscopy (I.J.B., with 20 years of experience). Surgical intervention was indicated for patients with persistent symptoms and positive clinical findings, with or without corresponding MRI abnormalities, following current clinical practice standards for FAIS management [18]. During arthroscopy, labral abnormalities were graded using the consensus-based classification system for intraoperative management of labral tears described by Domb et al [27], incorporating elements from Beck et al [28] and Lage et al [29] classifications. Furthermore, cartilage lesions were graded in each of the six distinct zones (A–F) as depicted in Fig. 1, according to the criteria established by the International Cartilage Research Society (ICRS) for the femoral cartilage [30] and according to the Haddad classification for the acetabular cartilage [31]. For comparison with MRI, intra-arthroscopic labral and cartilage findings were classified as present or absent. Details of the arthroscopic findings with ICRS/Haddad gradings from the patients included in this study are available in the supplementary material (S1).

### Statistical analysis

The statistical analysis was performed with SPSS, version 29.0 (IBM Corporation). Arthroscopy was set as reference of standard for all statistical comparisons, given its established role as the gold standard method for intra-articular hip pathology assessment. Sensitivity, specificity, and accuracy were calculated for detecting labral and cartilage defects overall, as well as for each cartilage zone and each reader individually. McNemar’s test was used to assess statistical significance for all comparisons, with *p*-values < 0.05 considered statistically significant. Interobserver agreement was evaluated using Gwet’s AC2 coefficient with 95% confidence intervals, accounting for the potential prevalence-bias in kappa statistics.

## Results

### Patient characteristics

A total of 32 patients with symptomatic FAIS were included in this study. The patient demographics and characteristics are summarized in Table 2. The mean age of the patients was 37.5 years (± 11.7), with a predominance of males (24 males, 8 females). The mean weight and height were 81 kg (± 15.3) and 178 cm (± 9.7), respectively. Clinical presentations included groin pain (*n* = 29, 91%), positive impingement test (*n* = 32, 100%), and limited internal rotation in 90° flexion. The mean symptom duration was 31.7 months (± 42.1). Surgeries were more frequently performed on the left side (59.4%) compared to the right side (40.6%).

**Table 2:** Patient characteristics

Patient characteristics	*N* = 32	Valid %
Age (years)*	37.5 (11.7)	
Female/male	8/24	25/75
Weight (kg)*	81 (15.3)	
Height (cm)*	178 (9.7)	
Groin pain	29	90.6
Positive impingement test	32	100
Symptom duration (months)*	31.7 (42.1)	
Side of surgery (left/right)	19/13	59.4/40.6

* Data is given as mean ± standard deviation

### Inter-rater reliability

Inter-rater reliability analysis (Table 3) demonstrated substantial agreement for labral abnormality detection (Gwet’s AC2 = 0.87 [95% CI: 0.77–0.97] for CS; 0.93 [95% CI: 0.85–1.00] for CSAI). For both CS and CSAI sequences, cartilage assessment revealed marked variability across anatomical zones. High inter-rater agreement was observed in peripheral acetabular zones for both sequences (Zone A: CS and CSAI = 0.94 [95% CI: 0.88–1.00]; Zone B: CS = 0.96 [95% CI: 0.89–1.00], CSAI = 0.92 [95% CI: 0.84–1.00]; Zone E: CS and CSAI = 0.96 [95% CI: 0.90–1.00]). Central acetabular zones demonstrated moderate to substantial agreement (Zone C: CS = 0.67 [95% CI: 0.49–0.85], CSAI = 0.80 [95% CI: 0.65–0.95]), while Zone D showed fair agreement (CS = 0.28 [95% CI: 0.07–0.49], CSAI = 0.39 [95% CI: 0.16–0.62]). Femoral cartilage assessment revealed similarly heterogeneous reliability, ranging from slight agreement in Zone C (CS = 0.08 [95% CI: 0.04–0.19], CSAI = 0.04 [95% CI: 0.05–0.12]) to excellent agreement in Zone E (CS and CSAI = 0.91 [95% CI: 0.80–1.00]). The low inter-rater reliability in femur zone C was due to the orthopedic surgeon not detecting any defect on the femur head.

**Table 3:** Frequency table and interobserver reliability

	Reader 1	Reader 2	Reader 3	Reader 4	Inter-rater reliability [95% CI]
Labrum					
CS	30/32 (94)	31/32 (97)	31/32 (97)	25/32 (78)	0.87 [0.77–0.97]
CSAI	30/32 (94)	31/32 (97)	31/32 (97)	28/32 (88)	0.93 [0.85–1.00]
Acetabulum zone A					
CS	1/32 (3)	3/32 (9)	0/32 (0)	0/32 (0)	0.94 [0.88–1.00]
CSAI	1/32 (3)	3/32 (9)	0/32 (0)	0/32 (0)	0.94 [0.88–1.00]
Acetabulum zone B					
CS	2/32 (6)	2/32 (6)	0/32 (0)	0/32 (0)	0.96 [0.89–1.00]
CSAI	4/32 (13)	2/32 (3)	0/32 (0)	1/32 (3)	0.92 [0.84–1.00]
Acetabulum zone C					
CS	27/32 (84)	28/32 (88)	27/32 (84)	23/32 (72)	0.67 [0.49–0.85]
CSAI	29/32 (91)	28/32 (88)	30/32 (94)	26/32 (81)	0.80 [0.65–0.95]
Acetabulum zone D					
CS	19/32 (59)	20/32 (63)	14/32 (44)	12/32 (38)	0.28 [0.07–0.49]
CSAI	20/32 (63)	20/32 (63)	22/32 (69)	15/32 (47)	0.39 [0.16–0.62]
Acetabulum zone E					
CS	1/32 (3)	2/32 (6)	0/32 (0)	0/32 (0)	0.96 [0.90–1.00]
CSAI	1/32 (3)	2/32 (6)	0/32 (0)	0/32 (0)	0.96 [0.90–1.00]
Femur zone A					
CS	15/32 (47)	15/32 (47)	3/32 (9)	0/32 (0)	0.47 [0.21–0.73]
CSAI	16/32 (50)	15/32 (47)	0/32 (0)	0/32 (0)	0.48 [0.22–0.75]
Femur zone B					
CS	4/32 (13)	5/32 (16)	1/32 (3)	1/32 (3)	0.86 [0.73–0.99]
CSAI	5/32 (16)	5/32 (16)	1/32 (3)	1/32 (3)	0.85 [0.71–0.99]
Femur zone C					
CS	24/32 (75)	24/32 (75)	21/32 (66)	0/32 (0)	0.08 [0.04–0.19]
CSAI	26/32 (81)	25/32 (78)	30/32 (94)	0/32 (0)	0.04 [0.05–0.12]
Femur zone D					
CS	8/32 (25)	8/32 (25)	22/32 (69)	0/32 (0)	0.32 [0.10–0.54]
CSAI	9/32 (25)	8/32 (28)	23/32 (72)	0/32 (0)	0.23 [0.00–0.46]
Femur zone E					
CS	4/32 (13)	4/32 (13)	0/32 (0)	0/32 (0)	0.91 [0.80–1.00]
CSAI	4/32 (13)	4/32 (13)	0/32 (0)	0/32 (0)	0.91 [0.80–1.00]

### Overall performance for all readers

The diagnostic performance of MR imaging using both standard-resolution CS and high-resolution CSAI sequences was assessed. Both CSAI and CS sequences exhibited high sensitivity (CS: 94% [95% CI: 90.3–98.4]; CSAI: 97% [95% CI: 93.7–99.9; *p* = 0.08]) and specificity (both CS and CSAI: 100%) for detecting labral abnormalities with no significant difference.

The overall sensitivity for detecting cartilage lesions was comparatively low, though overall sensitivity across all cartilage zones was significantly higher in CSAI compared to CS (CS: 37% [95% CI: 33.6–41.8]; CSAI 42% [95% CI: 37.5–45.9]; *p* < 0.001) and overall sensitivity across all acetabulum zones was significantly higher in CSAI vs. CS (CS: 39% [95% CI: 34.5–43.6]; CSAI: 44% [95% CI: 39.4–48.6]; *p* < 0.001). Averaged for all readers, the highest sensitivity for assessment of cartilage defects is in the superolateral zone, where CSAI showed significantly higher sensitivity compared to CS (CS: 81% [95% CI: 74.5–88.0]; CSAI: 88% [95% CI: 82.7–93.9]; *p* < 0.05). It is noteworthy that all patients presented with superolateral to anterosuperior acetabular defects (zones C and D), precluding specificity calculations for these zones due to the absence of true negative cases. Particularly low averaged sensitivity was found for the assessment of the anteroinferior- and posterior zones of the acetabulum, as well as the inferior and posterior zones of the femur (both CS and CSAI < 6%).

Overall specificity showed a small but significant decrease from CS to CSAI sequences (CS: 81% [95% CI: 78.6–84.2]; CSAI: 79% [95% CI: 76.4–82.2]; *p* < 0.001) and overall specificity across all femur zones was significantly lower in CSAI vs. CS (CS: 76% [95% CI: 72.4–79.4]; CSAI 73% [95% CI: 69.7–77.1]; *p* < 0.001). The highest specificity was in the assessment of the anteroinferior acetabular zone (both CS and CSAI: 99%). The lowest average specificity was observed in the assessment the femoral weight-bearing zone (Zone C), with specificity significantly lower in CSAI compared to CS (CS: 47% [95% CI: 37.8–56.6]; CSAI: 37% [95% CI: 27.9–46.1]; *p* < 0.001). Detailed results are presented in Table 4.

**Table 4:** Overall performance evaluation, averaged for all four readers

	Overall					
	CS			CSAI		
	Sensitivity (%) [95% CI]	Specificity (%) [95% CI]	Accuracy (%) [95% CI]	Sensitivity (%) [95% CI]	Specificity (%) [95% CI]	Accuracy (%) [95% CI]
Labrum	94.4 [90.3–98.4]	100 [100–100]	94.5 [94.9–100]	96.8 [93.7–99.9]	100 [100–100]	96.9 [93.9–99.9]
Acetabulum zone A	0.0 [0.0–0.0]	95.2 [90.7–99.8]	62.5 [54.1–70.9]	0.0 [0.0–0.0]	95.2 [90.7–99.8]	62.5 [54.1–70.9]
Acetabulum zone B	4.5 [0.2–8.9]	100 [100–100]	34.3 [26.1–42.6]	5.7 [0.8–10.5]	95.0 [88.2–100]	33.6 [25.4–41.8]
Acetabulum zone C	81.3 [74.5–88.0]	-	81.3 [74.5–88.0]	88.3 [82.7–93.9]*	-	88.3 [82.7–93.9]*
Acetabulum zone D	50.8 [42.1–59.4]	-	50.8 [42.1–59.4]	60.2 [51.7–68.6]**	-	60.2 [51.7–68.6]**
Acetabulum zone E	3.3 [0.0–7.9]	98.5 [95.7–100]	53.9 [45.3–62.5]	3.3 [0.0–7.9]	98.5 [95.7–100]	53.9 [45.3–62.5]
Femur zone A	31.3 [8.5–54.0]	75.0 [67.0–83.0]	69.5 [61.6–77.5]	25.0 [3.8–46.2]	75.9 [68.0–83.8]	69.5 [61.6–77.5]
Femur zone B	0.0 [0.0–0.0]	90.5 [85.2–95.8]	93.8 [75.4–88.7]	0.0 [0.0–0.0]	89.7 [84.1–95.2]	81.2 [74.5–88.0]
Femur zone C	60.0 [38.5–81.5]	47.2 [37.8–56.6]	49.2 [40.6–57.9]	65.0 [44.1–85.9]	37.0 [27.9–46.1]**	41.4 [32.9–49.9]*
Femur zone D	25.0 [6.0–44.0]	69.4 [60.8–78.1]	62.5 [54.1–70.9]	20.0 [2.5–37.5]	66.7 [47.7–64.8]	59.4 [50.9–67.9]*
Femur zone E	1.7 [0.0–37.8]	94.8 [90.8–98.9]	87.5 [81.8–93.2]	1.7 [0.0–37.8]	94.8 [90.8–98.9]	87.5 [81.8–93.2]
All cartilage zones	37.0 [33.6–41.8]	81.4 [78.6–84.2]	63.4 [60.7–66.0]	41.7 [37.5–45.9]**	79.3 [76.4–82.2]**	63.8 [61.1–66.4]*
All acetabulum zones	39.0 [34.5–43.6]	97.4 [95.1–99.6]	56.6 [52.7–60.4]	44.0 [39.4–48.6]**	96.3 [93.7–99.0]	59.7 [55.9–63.5]**
All femur zones	30.0 [20.0–40.0]	75.9 [72.4–79.4]	70.0 [66.6–73.7]	28.8 [18.8–38.7]	73.4 [69.7–77.1]**	67.8 [64.2–71.4]**

McNemar’s test was used to compare sensitivity, specificity, and accuracy between CS and CSAI sequences. Statistically significant differences are indicated in the CSAI column

*p* < 0.05 (*) and *p* < 0.001 (**) denote significant and highly significant differences, respectively

### Individual performance evaluation

**Fig. 2:**
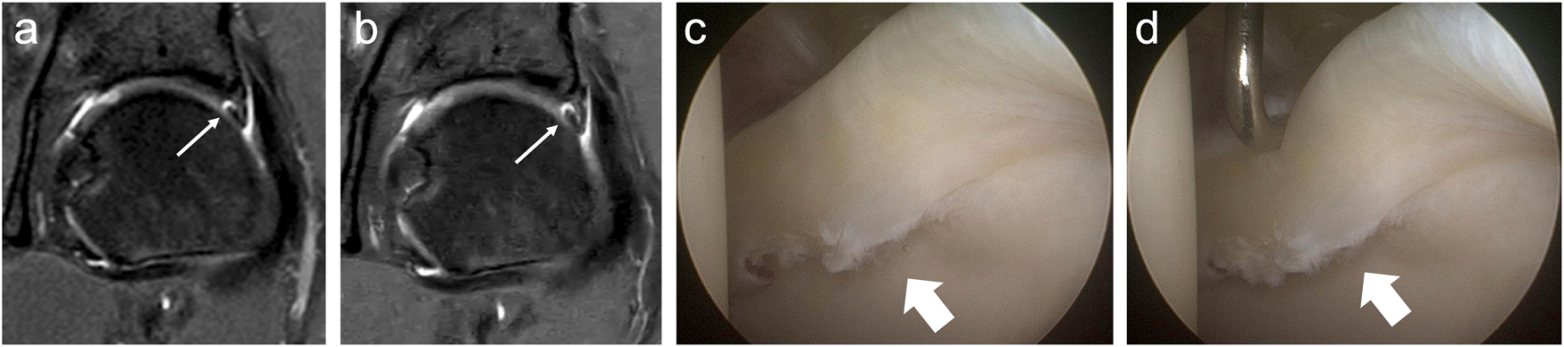
True positive (left hip). MR: Coronal IM-weighted TSE images of the left hip show a labral lesion (arrow) in the CS (**a**) and CSAI (**b**) sequences. Arthroscopy: **c** A full-thickness labral tear (thick arrow) with chondrolabral separation at the junctional zone. **d** Arthroscopic probing confirms an unstable labral tear (thick arrow)

The performance of individual readers in detecting labral abnormalities and cartilage defects on MR imaging versus arthroscopy is summarized in Table 5. All readers demonstrated high sensitivity and specificity in assessing labral abnormalities, with values as high as 100% for both CS and CSAI. The expert orthopedic surgeon improved sensitivity for labral abnormalities from 81% with CS to 90% with CSAI; however, this difference did not reach statistical significance (*p* = 0.08). Figure 2 illustrates an instance of a labral defect.

**Table 5:** Individual performance evaluation for all four readers

	Resident					
	CS			CSAI		
	Sensitivity	Specificity	Accuracy	Sensitivity	Specificity	Accuracy
Labrum	96.8% (30/31)	100% (1/1)	96.9% (31/32)	96.8% (30/31)	100% (1/1)	96.9% (31/32)
Acetabulum zone A	0.0% (0/11)	95.2% (20/21)	62.5% (20/32)	0.0% (0/11)	95.2 (20/21)	62.5% (20/32)
Acetabulum zone B	9.1% (2/22)	100% (10/10)	37.5% (12/32)	9.1% (2/22)	80.0% (8/10)	31.3% (10/32)
Acetabulum zone C	84.4% (27/32)	-	84.4% (27/32)	90.6% (29/32)	-	90.6% (29/32)
Acetabulum zone D	59.4% (19/32)	-	59.4% (19/32)	62.5% (20/32)	-	62.5% (20/32)
Acetabulum zone E	6.7% (1/15)	100% (17/17)	56.3% (18/32)	6.7% (1/15)	100% (17/17)	56.3% (18/32)
Femur zone A	50.0% (2/4)	53.6% (15/28)	53.1% (17/32)	50.0% (2/4)	50.0% (14/28)	50.0% (16/32)
Femur zone B	0.0% (0/3)	86.2% (25/29)	78.1% (25/32)	0.0% (0/3)	82.8% (24/29)	75.0% (24/32)
Femur zone C	80.0% (4/5)	25.9% (7/27)	34.4% (11/32)	80.0% (4/5)	18.5% (5/27)	28.1% (9/32)
Femur zone D	20.0% (1/5)	74.1% (20/27)	65.6% (21/32)	20.0% (1/5)	70.4% (19/27)	62.5% (20/32)
Femur zone E	33.3% (1/3)	89.7% (26/29)	84.4% (27/32)	33.3% (1/3)	89.7% (26/29)	84.4% (27/32)
All cartilage zones	43.2% (57/132)	74.4% (140/188)	61.6% (197/320)	45.5% (60/132)	70.7% (133/188)*	60.3% (193/320)*
All acetabulum zones	43.6% (49/112)	97.9% (47/48)	60.0% (96/160)	46.4% (52/112)	93.8% (45/48)	60.6% (97/160)
All femur zones	40.0% (8/20)	66.4% (93/140)	63.1% (101/160)	40.0% (8/20)	62.9% (88/140)*	60.0% (96/160)*

	Musculoskeletal (MSK) Fellow					
	CS			CSAI		
	Sensitivity	Specificity	Accuracy	Sensitivity	Specificity	Accuracy
Labrum	100% (31/31)	100% (1/1)	100% (32/32)	100% (31/31)	100% (1/1)	100% (32/32)
Acetabulum zone A	0.0% (0/11)	85.7% (18/21)	56.3% (18/32)	0.0% (0/11)	85.7% (18/21)	56.3% (18/32)
Acetabulum zone B	9.1% (2/22)	100% (10/10)	37.5% (12/32)	9.1% (2/22)	100% (10/10)	37.5% (12/32)
Acetabulum zone C	87.5% (28/32)	-	87.5% (28/32)	87.5% (28/32)	-	87.5% (28/32)
Acetabulum zone D	62.5% (20/32)	-	62.5% (20/32)	62.5% (20/32)	-	62.5% (20/32)
Acetabulum zone E	6.7% (1/15)	94.1% (16/17)	53.1% (17/32)	6.7% (1/15)	94.1% (16/17)	53.1% (17/32)
Femur zone A	50.0% (2/4)	53.6% (15/28)	53.1% (17/32)	50.0% (2/4)	53.6% (15/28)	53.1% (17/32)
Femur zone B	0.0% (0/3)	82.8% (24/29)	75.0% (24/32)	0.0% (0/3)	82.8% (24/29)	75.0% (24/32)
Femur zone C	80.0% (4/5)	25.9% (7/27)	34.4% (11/32)	80.0% (4/5)	22.2% (6/27)	31.3% (10/32)
Femur zone D	20.0% (1/5)	74.1% (20/27)	65.6% (21/32)	20.0% (1/5)	74.1% (20/27)	65.6% (21/32)
Femur zone E	33.3% (1/3)	89.7% (26/29)	84.4% (27/32)	33.3% (1/3)	89.7% (26/29)	84.4% (27/32)
All cartilage zones	44.7% (59/132)	72.3% (136/188)	60.9% (195/320)	44.7% (59/132)	71.8% (135/188)	60.6% (194/320)
All acetabulum zones	45.6% (51/112)	91.7% (44/48)	59.4% (95/160)	45.5% (51/112)	91.7% (44/48)	59.4% (95/160)
All femur zones	40.0% (8/20)	65.7% (92/140)	62.5% (100/160)	40.0% (8/20)	65.0% (91/140)	61.9% (99/160)

	Fellowship-trained MSK radiologist					
	CS			CSAI		
	Sensitivity	Specificity	Accuracy	Sensitivity	Specificity	Accuracy
Labrum	100% (31/31)	100% (1/1)	100% (32/32)	100% (31/31)	100% (1/1)	100% (32/32)
Acetabulum zone A	0.0% (0/11)	100% (21/21)	65.5% (21/32)	0.0% (0/11)	100% (21/21)	65.6% (21/32)
Acetabulum zone B	0.0% (0/22)	100% (10/10)	31.3% (10/32)	0.0% (0/22)	100% (10/10)	31.3% (10/32)
Acetabulum zone C	84.4% (27/32)	-	84.4% (27/32)	93.8% (30/32)	-	93.8% (30/32)
Acetabulum zone D	43.8% (14/32)	-	43.8% (14/32)	68.8% (22/32)*	-	68.8% (22/32)*
Acetabulum zone E	0.0% (0/15)	100% (17/17)	53.1% (17/32)	0.0% (0/15)	100% (17/17)	53.1% (17/32)
Femur zone A	25.0% (1/4)	100% (26/28)	84.4% (27/32)	0.0% (0/4)	100% (28/28)	87.5% (28/32)
Femur zone B	0.0% (0/3)	96.6% (28/29)	87.5% (28/32)	0.0% (0/3)	96.6% (28/29)	87.5% (28/32)
Femur zone C	80.0% (4/5)	37.0% (10/27)	43.8% (14/32)	100.0% (5/5)	7.4% (2/27)*	21.9% (7/32)*
Femur zone D	60.0% (3/5)	29.6% (8/27)	34.4% (11/32)	40.0% (2/5)	22.2% (6/27)	25.0% (8/32)
Femur zone E	0.0% (0/3)	100% (29/29)	90.6% (29/32)	0.0% (0/3)	100% (29/29)	90.6% (29/32)
All cartilage zones	37.1% (49/132)	79.3% (149/188)	61.9% (198/320)	44.7% (59/132)*	75.0% (141/188)*	62.5% (200/320)
All acetabulum zones	36.6% (41/112)	100% (48/48)	55.6% (89/160)	46.4% (52/112)**	100% (48/48)	62.5% (100/160)**
All femur zones	40.0% (8/20)	72.1% (101/140)	68.1% (109/160)	35.0% (7/20)	66.4% (93/140)*	62.5% (100/160)*

	Orthopedic Surgeon					
	CS			CSAI		
	Sensitivity	Specificity	Accuracy	Sensitivity	Specificity	Accuracy
Labrum	80.6% (25/31)	100% (1/1)	81.3% (26/32)	90.3% (28/31)	100% (1/1)	90.6% (29/32)
Acetabulum zone A	0.0% (0/11)	100% (21/21)	65.5% (21/32)	0.0% (0/11)	100% (21/21)	65.6% (21/32)
Acetabulum zone B	0.0% (0/22)	100% (10/10)	31.3% (10/32)	4.5% (1/22)	100% (10/10)	34.4% (11/32)
Acetabulum zone C	71.9% (23/32)	-	71.9% (23/32)	81.3% (26/32)	-	81.3% (26/32)
Acetabulum zone D	37.5% (12/32)	-	37.5% (12/32)	46.9% (15/32)	-	46.9% (15/32)
Acetabulum zone E	0.0% (0/15)	100% (17/17)	53.1% (17/32)	0.0% (0/15)	100% (17/17)	53.1% (17/32)
Femur zone A	0.0% (0/4)	100% (28/28)	87.5% (28/32)	0.0% (0/4)	100% (28/28)	87.5% (28/32)
Femur zone B	0.0% (0/3)	96.6% (28/29)	87.5% (28/32)	0.0% (0/3)	96.6% (28/29)	87.5% (28/32)
Femur zone C	0.0% (0/5)	100% (27/27)	84.4% (27/32)	0.0% (0/5)	100% (27/27)	84.4% (27/32)
Femur zone D	0.0% (0/5)	100% (27/27)	84.4% (27/32)	0.0% (0/5)	100% (27/27)	84.4% (27/32)
Femur zone E	0.0% (0/3)	100% (29/29)	90.6% (29/32)	0.0% (0/3)	100% (29/29)	90.6% (29/32)
All cartilage zones	26.5% (35/132)	99.5% (187/188)	69.4% (222/320)	31.8% (42/132)*	99.5% (187/188)	71.6% (229/320)*
All acetabulum zones	31.3% (35/112)	100% (48/48)	51.9% (83/160)	37.5% (42/112)*	100% (48/48)	56.3% (90/160)*
All femur zones	0.0% (0/20)	99.3% (139/140)	86.9% (139/160)	0.0% (0/20)	99.3% (139/140)	86.9% (139/160)

Readers were blinded to all clinical findings

McNemar’s test was used to compare sensitivity, specificity, and accuracy between CS and CSAI sequences. Statistically significant differences are indicated in the CSAI column

*p* < 0.05 (*) and *p* < 0.001 (**) denote significant and highly significant differences, respectively

**Fig. 3:**
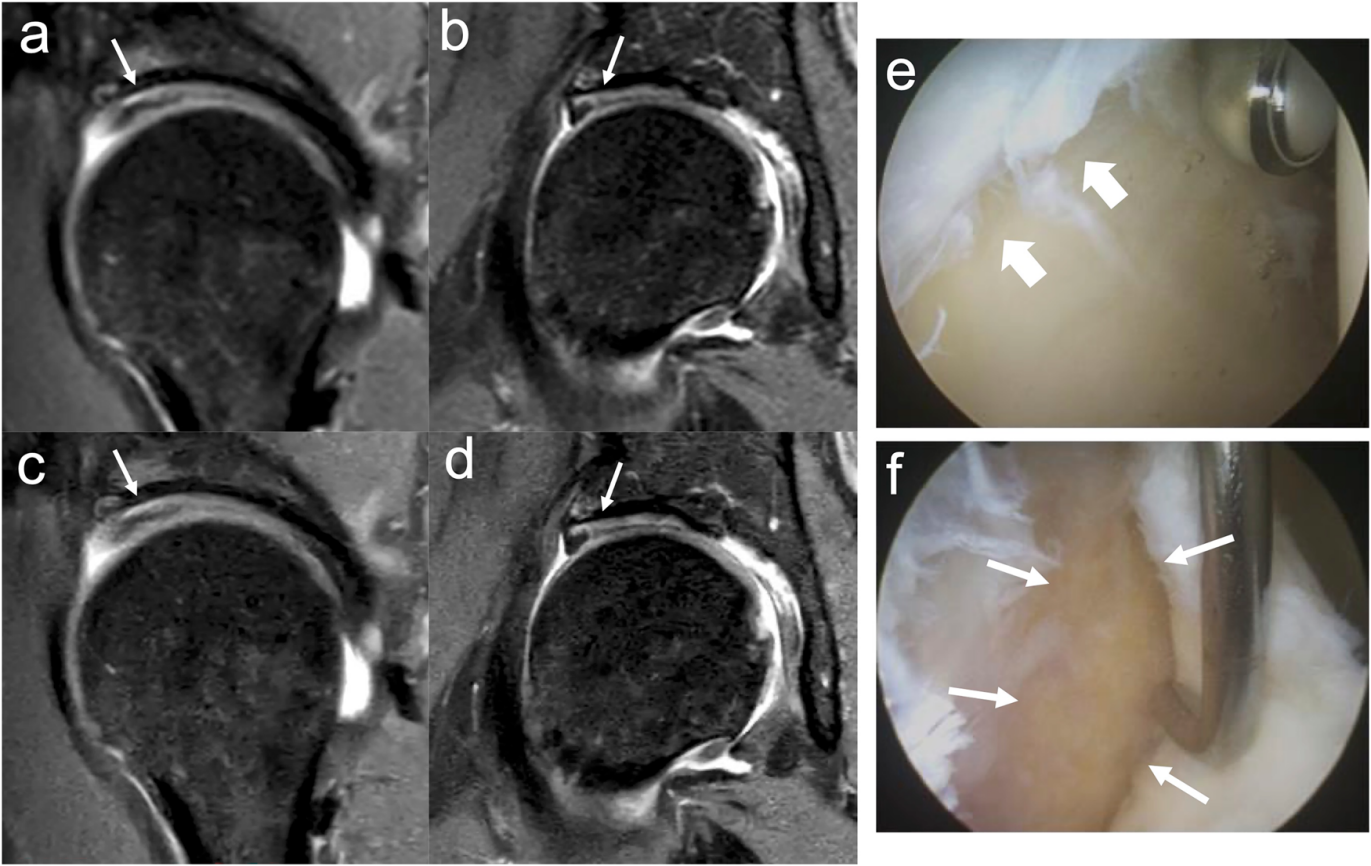
True positive (right hip). MR: CS (**a**—sagittal, **b**—coronal) and CSAI (**c**—sagittal, **d**—coronal) IM-weighted TSE images both demonstrate a chondrolabral defect in acetabular zone C (arrows). Arthroscopy: **e** Delamination of the articular cartilage with macroscopic debonding (thick arrows) and (**f**) exposure of the acetabular bone (arrows), confirmed by arthroscopic probing (Haddad grade 4)

**Fig. 4:**
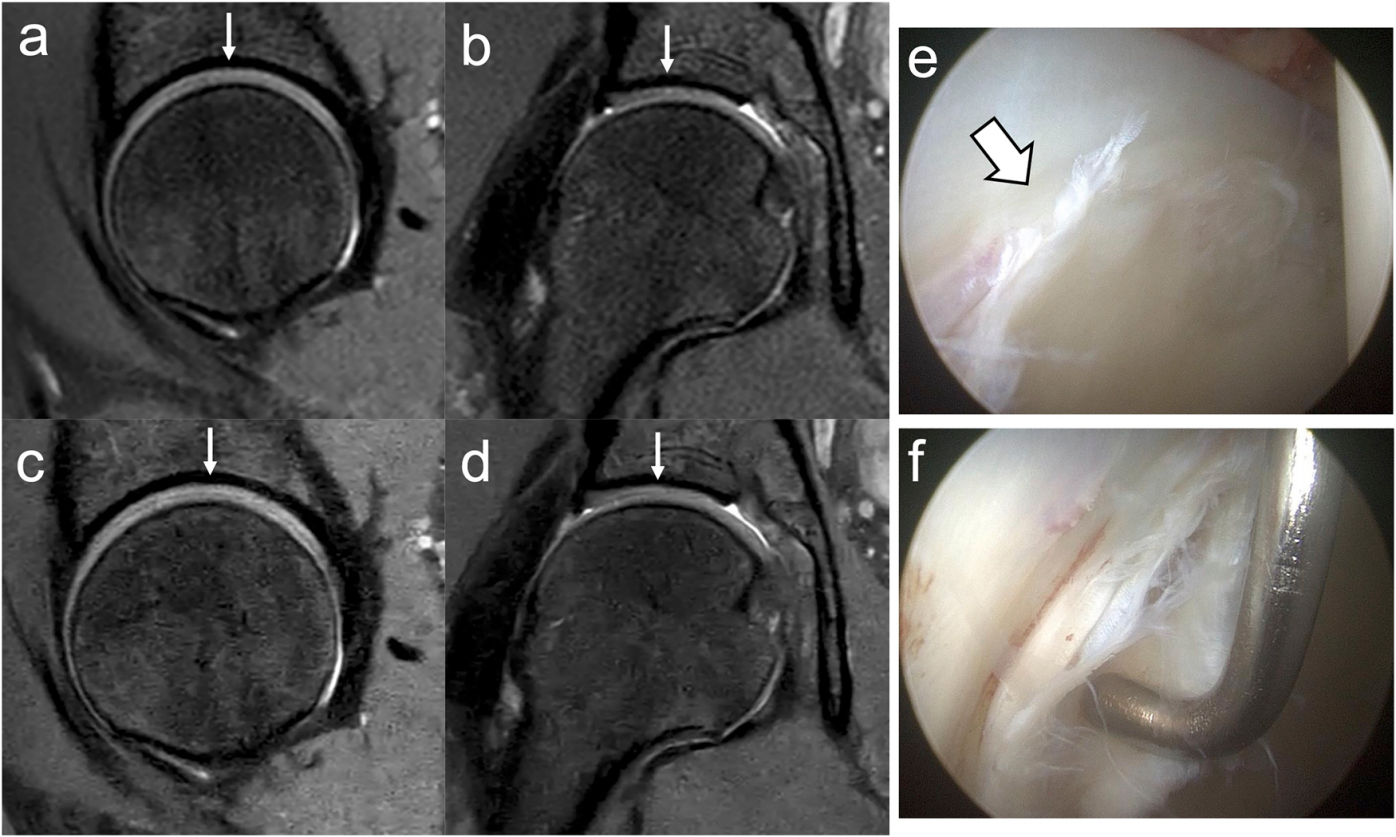
False negative (right hip). MR: IM-weighted TSE images with CS (**a**—sagittal, **b**—coronal) and CSAI (**c**—sagittal, **d**—coronal). There is no detectable pathology in acetabular zone C. The arrows are pointing to acetabular zone C. Arthroscopy: **e** Cleavage tear (thick arrow) with separation at the chondrolabral junction, surface roughening, and fibrillation of the cartilage. **f** Arthroscopic probing reveals adherence of the articular cartilage to the subchondral bone, with no delamination detected (Haddad grade 2)

The sensitivity for evaluating acetabular cartilage was predominantly poor in Zones A (0%) and B (0–9%), but it increased in Zones C and D, reaching up to 94% and 63%, respectively. Figure 3 illustrates a true positive cartilage defect in acetabular zone C, while Fig. 4 depicts a false negative. The fellowship-trained MSK radiologist demonstrated statistically significant improvements with CSAI, including increased sensitivity in acetabular Zone D (CS: 44%; CSAI: 69%; *p* < 0.05) and overall acetabular zones (CS: 37%; CSAI: 46%; *p* < 0.001). The orthopedic surgeon also showed significant improvement in overall cartilage sensitivity with CSAI compared to CS (CS: 27%; CSAI: 32%; *p* < 0.05), particularly in acetabular zones (CS: 32%; CSAI: 38%; *p* < 0.05).

**Fig. 5:**
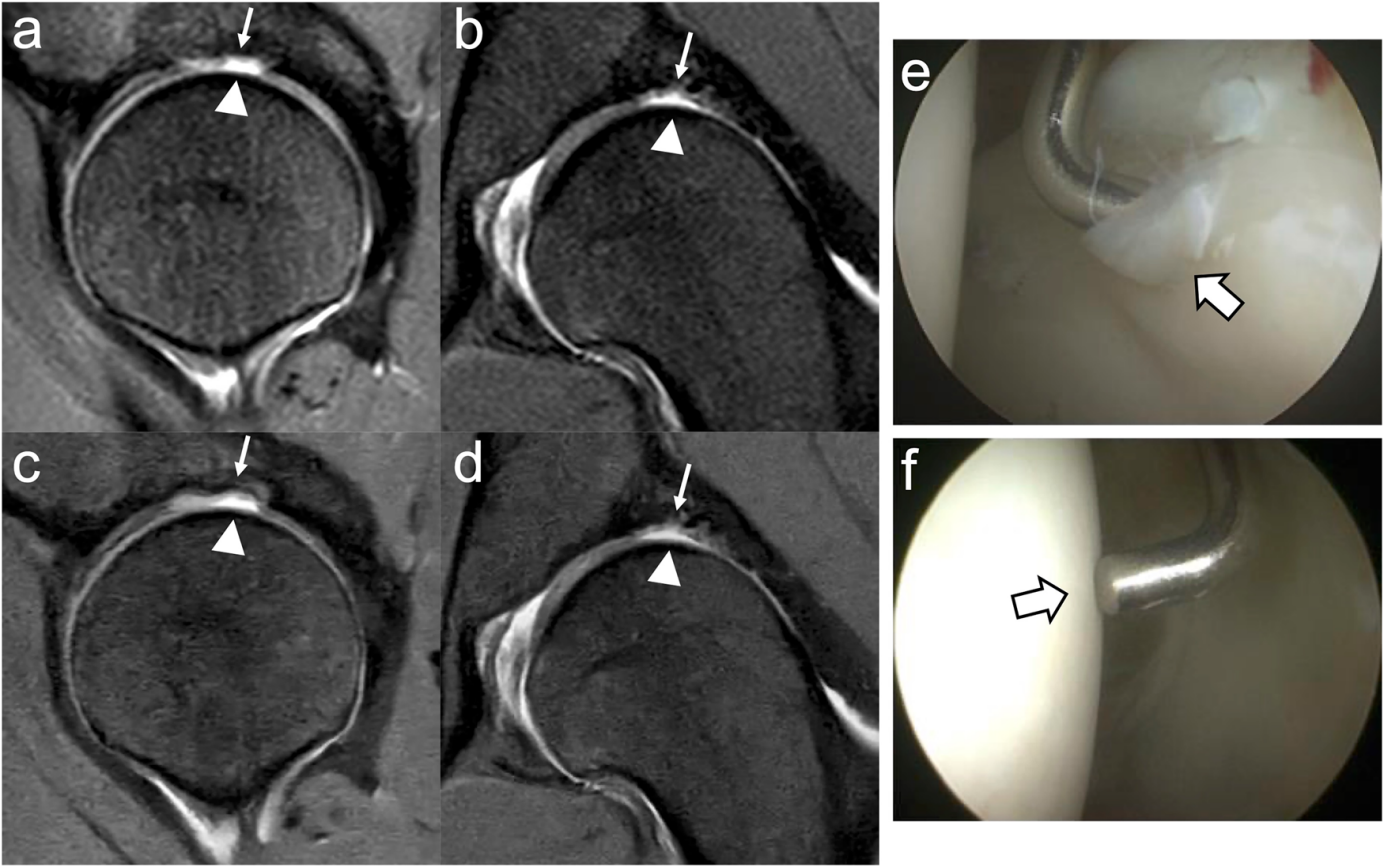
False positive for femur zone C (left hip). MR: IM-weighted TSE images with CS (**a**—sagittal, **b**—coronal) and CSAI (**c**—sagittal, **d**—coronal). Readers detected a chondrolabral defect on acetabular zone C (arrows) with a ‘kissing-lesion’ on femoral cartilage zone C (arrow heads). Arthroscopy: **e** Separation at the chondrolabral junction with cartilage delamination (thick arrow), confirmed by probing (Haddad grade 3). **f** No arthroscopic evidence of femoral head cartilage damage (thick arrow)

For the radiologists the sensitivity in the assessment of femoral cartilage defects was inconsistent, with sensitivity ranging from 0 to 50% in Zones A and B and up to 100% in Zone C. Only the fellowship-trained MSK radiologist demonstrated a non-significant improvement in sensitivity in femoral Zone C from 80% in CS to 100% CSAI, albeit a with significantly decreased specificity (CS: 37%; CSAI: 7%; *p* < 0.05). Figure 5 illustrates an instance of a false positive cartilage defect in the femoral cartilage zone C. The orthopedic surgeon identified no abnormalities in the femoral cartilage.

## Discussion

The present study evaluated the diagnostic performance of high-resolution deep learning-based CSAI sequences compared to standard-resolution CS sequences for detecting labral and cartilage defects in the hip joint, using hip arthroscopy as the reference standard. Readers interpreting CS and CSAI MR images demonstrated excellent results for diagnosing labral abnormalities, however the detection of cartilage lesions remained limited, even with high-resolution protocols.

In accordance with our study, Neumann et al reported a high sensitivity of 92.5% for the detection of labral defects on non-arthrographic MR imaging [32], while Ibrahimi et al reported a sensitivity of 100% [33], both studies including patients with FAIS and using hip arthroscopy as standard of reference. However, given that 31 out of 32 patients in our cohort and all patients in the studies by Neumann et al and Ibrahimi et al presented with labral tears, the ability to draw reliable conclusions regarding specificity is inherently limited. Previous results for detecting cartilage lesions have been more variable, with Ibrahimi et al reporting a sensitivity of 60%, while Neumann et al found a sensitivity of 96% [32, 33]. A meta-analysis demonstrated a pooled sensitivity and specificity of 59% and 94% for non-arthrographic MR imaging [34].

While substantial inter-rater reliability was observed for labral assessment (Gwet’s AC2: 0.87–0.93), marked variability was noted in cartilage evaluation across different zones. Notably, the lower inter-rater reliability observed in femoral Zone C (Gwet’s AC2: 0.04–0.08) could be attributed to the differences in cartilage assessment between radiological readers and orthopedic surgeons rather than sequence-specific limitations. These findings underscore the inherent complexity of cartilage signal interpretation in MR imaging, independent of the acquisition technique employed.

In our study cohort, the diagnostic performance was highest for chondral abnormalities in zones C and D, which represent the superior and anterosuperior zones of the acetabulum and the corresponding femoral zones. Notably, these zones demonstrated universal presence of cartilage defects at arthroscopy, precluding specificity calculations due to the absence of true negative cases. Cartilage damage in the hip typically occurs at the acetabulum, primarily at the superolateral to anterosuperior facet (zones C/D), and is frequently associated with FAIS [23, 35]. Detecting damage to the femoral head cartilage presents significant challenges attributed to anatomical factors, with normal cartilage thickness varying from 0.32 to 2.53 mm at the femoral head and 0.95 to 3.13 mm at the acetabulum [7]. Isolated damage to the femoral head cartilage is uncommon, usually resulting from trauma or avascular necrosis. When bilateral cartilage damage is evident, affecting both the acetabulum and the femoral head, it usually indicates, that hip degeneration is already significantly advanced (Tönnis grade > 1–2 osteoarthritis) [36, 37]. In these instances, the success rates of arthroscopic treatment for FAIS are typically less effective, emphasizing the importance of accurate preoperative assessment of the acetabular cartilage damage.

Recent advancements in imaging technology, including deep learning algorithms and compressed sense sequences, have enhanced image quality and facilitated the identification of abnormalities by radiologists. For instance, Dratsch et al demonstrated the efficacy of deep learning-enhanced MR imaging of the shoulder, indicating potential applications for other joints, such as the hip [14]. Similarly, 3D MR imaging has been shown to improve the visualization of anatomical structures by providing isotropic resolution and high-quality multiplanar reconstructions [38, 39]. This capability is particularly beneficial for complex joints like the hip, where intricate spatial relationships are critical. Additionally, 3D MR imaging has shown to improve the detection of subtle labral and cartilage abnormalities [40, 41]. However, its clinical application is limited by motion artifacts, which remain a significant challenge [39, 41].

Complementary imaging techniques, such as traction MR imaging and MR arthrography are often employed for preoperative evaluation of patients with FAIS. Schmaranzer et al reported that readers achieved an accuracy of 92% in detecting femoroacetabular cartilage defects when interpreting traction MR images [42]. MR arthrography has been shown to improve the detection of labral and cartilage abnormalities. However, a meta-analysis demonstrated comparable diagnostic performance between non-arthrographic MR imaging and MR arthrography, with pooled sensitivity and specificity of 59% and 94% for non-arthrographic MR imaging in detecting cartilage lesions, compared to 62% and 86% for MR arthrography [34]. Given that MR arthrography is more time-consuming and invasive, with some patients experiencing post-procedural discomfort, the choice between conventional MR imaging and MR arthrography should be based on institutional protocols and the optimization of scanning techniques to balance diagnostic efficacy and patient comfort.

This study has several limitations. The small sample size and predominance of patients with labral defects limit the generalizability of our findings. Additionally, no complementary imaging techniques such as traction MR imaging or intra-articular contrast were included, which may have reduced the diagnostic performance to detect subtle cartilage lesions. Also, we did not correlate different severity grades of cartilage lesions with arthroscopic ICRS and Haddad gradings, primarily due to the limited sample size and evidence from prior studies suggesting that grading comparisons may lower diagnostic accuracy [43, 44]. This leaves a gap in understanding the diagnostic performance across varying stages of cartilage damage. Future studies should involve larger, more diverse patient populations and could potentially include complementary techniques, such as traction MR imaging, to enhance diagnostic capabilities. Furthermore, the arthroscopic surgeon’s prior knowledge of MRI findings represents a potential verification bias, although this reflects real-world clinical practice where preoperative imaging guides surgical approach and technique. Future studies might consider blinding the operating surgeon to specific MRI findings while maintaining access to essential preoperative planning information.

In conclusion, readers interpreting CSAI and CS MR images demonstrated excellent accuracy in identifying labral abnormalities. CSAI imaging demonstrated improved overall cartilage sensitivity compared to CS, particularly in acetabular zones. However, this enhanced sensitivity was counterbalanced by decreased specificity in certain anatomical regions, notably in the femoral weight-bearing zone. The overall diagnostic performance for cartilage lesions remained suboptimal. These results highlight the current limitations of conventional MR imaging for evaluating cartilage abnormalities, even with optimized high-resolution sequences. Further technical developments and investigations are warranted to improve the diagnostic performance.

## Supplementary information

ELECTRONIC SUPPLEMENTARY MATERIAL

## Abbreviations

CNN: Convolutional neural network
CS: Compressed sensing
CSAI: Reconstruction algorithm, combining parallel imaging, compressed sensing and deep learning
FAIS: Femoroacetabular impingement syndrome
ICRS: International Cartilage Research Society
IM: Intermediate-weighted
MR: Magnetic resonance
MSK: Musculoskeletal
SPIR: Spectral presaturation with inversion recovery
TSE: Turbo-spin-echo
