# Supplementary material for: Deep learning and conventional hip MRI for the detection of labral and cartilage abnormalities using arthroscopy as standard of reference
Source: Eur Radiol. 2025 Apr 16;35(10):6065–78. doi: 10.1007/s00330-025-11546-9 (PMC12417240; doi:10.1007/s00330-025-11546-9)
Supplement: Supplementary file 1 — ELECTRONIC SUPPLEMENTARY MATERIAL [file 330_2025_11546_MOESM1_ESM.pdf]

Deep Learning and conventional Hip MRI for the Detection of Labral and Cartilage Abnormalities using Arthroscopy as Standard of Reference

ELECTRONIC SUPPLEMENTARY MATERIAL

Supplementary 1: Arthroscopic Findings

	Grade	*n*
**Acetabulum (Haddad)**		
Zone A	I	
	II	
	III	2
	IV	9
Total		11
Zone B	I	
	II	1
	III	9
	IV	12
Total		22
Zone C	I	
	II	1
	III	17
	IV	14
Total		32
Zone D	I	
	II	1
	III	18
	IV	13
Total		32
Zone E	I	
	II	
	III	4
	IV	11
Total		15
**Femur (ICRS)**		
Zone A	I	
	II	
	III	4
	IV	
Total		4
Zone B	I	
	II	
	III	3
	IV	
Total		3
Zone C	I	
	II	
	III	4
	IV	1
Total		5
Zone D	I	
	II	
	III	4
	IV	1
Total		5
Zone E	I	
	II	
	III	3
	IV	
Total		3
Total labral defects		31
